# Monitoring Double-Cropped Extent with Remote Sensing in Areas with High Crop Diversity

**DOI:** 10.3390/plants14091362

**Published:** 2025-04-30

**Authors:** Hossein Noorazar, Michael P. Brady, Supriya Savalkar, Amin Norouzi Kandelati, Mingliang Liu, Perry Beale, Andrew M. McGuire, Timothy Waters, Kirti Rajagopalan

**Affiliations:** 1 Department of Biological Systems Engineering, Washington State University, Pullman, WA 99164, USA; 2 School of Economic Sciences, Washington State University, Pullman, WA 99164, USA; 3 Department of Civil and Environmental Engineering, Washington State University, Pullman, WA 99352, USA; 4 Washington State Department of Agriculture, Yakima, WA 98504, USA; 5 Agriculture and Natural Resources Extension Program, Washington State University, Moses Lake, WA 98837, USA; 6 Agriculture and Natural Resources Extension Program, Washington State University, Pasco, WA 99301, USA

**Keywords:** double-/multi-cropping, cropping intensity, Landsat, NDVI, remote sensing, machine learning

## Abstract

The extent of single- and multi-cropping systems in any region, as well as potential changes to them, has consequences on food security and land- and water-resource use, raising important management questions. However, addressing these questions is limited by a lack of reliable data on multi-cropping practices at a high spatial resolution, especially in areas with high crop diversity. In this paper, we develop and apply a relatively low-cost and scalable method to identify double-cropping at the field scale using satellite (Landsat) imagery. The process combines machine learning methods with expert labeling. The process evaluates multiple machine learning methods, including an image classification of a time-series, trained on data where cropping intensity labels were created by experts who are familiar with regional production practices. We demonstrate the process by measuring double-cropping extent in a part of Washington State in the Pacific Northwest United States—an arid region with cold winters and hot summers with significant production of more than 60 distinct types of crops including hay, fruits, vegetables, and grains in irrigated settings. Our results indicate that the current state-of-the-art methods for identifying cropping intensity—which apply simpler rule-based thresholds on vegetation indices—do not work well in regions with a high crop diversity and likely significantly overestimate double-cropped extent. Multiple machine learning models were applied on Landsat-derived vegetation index time-series data and were able to perform better by capturing nuances that the simple rule-based approaches are unable to. In particular, our (image-based) deep learning model was able to capture nuances in this crop-diverse environment and achieve a high accuracy (96–99% overall accuracy and 83–93% producer accuracy for the double-cropped class with a standard error of less than 2.5%) while also identifying double-cropping in the right crop types and locations based on expert knowledge. Our expert labeling process worked well and has potential as a relatively low-cost, scalable approach for remote sensing applications. The product developed here is valuable for the long-term monitoring of double-cropped extent and for informing several policy questions related to food production and resource use.

## 1. Introduction

Spatially explicit characterization of land cover and land use, as well as their changes over time, is key to long-term agricultural, environmental, and natural resource planning and management. One land-use characteristic that is becoming increasingly relevant for a wide range of policy questions is the extent of single-cropping versus multi-cropping systems; the latter refers to multiple crops grown and harvested in the same field in the same year. For example, government-subsidized insurance for farmers that practice multi-cropping has been proposed as a way to increase production and keep food prices down [1]. Additionally, climate change has the potential to disrupt single-cropping systems in positive and negative ways. On the positive side, longer growing seasons under warming can allow cooler regions that historically could only support single-cropping to transition into multi-cropping systems [2], increase total food production, and make farming more profitable. On the negative side, such a switch can increase crop irrigation water demands, exacerbating water security concerns [3]. It may also increase fertilizer use, exacerbating nutrient loading problems in water bodies and negatively affecting soil health in general [4,5]. Therefore, having spatio-temporal information on the prevalence of multi-cropping is important for understanding these implications, but such data are lacking. Even in the United States (US), multi-cropping practices are surveyed only for a few grain commodity crops [6], and this information is not available for areas with a large crop diversity.

Over the last decade, several studies have utilized satellite imagery and mapped cropping intensities, where intensities of 1, 2, and 3 are indicative of single-, double-, and triple-cropping, respectively. While earlier work utilized coarse-spatial-resolution imagery at 0.5 to several-kilometer resolutions [7,8,9,10], recent work has focused on Landsat or Sentinel data at finer spatial resolutions of 10 m to 30 m [11,12,13,14]. With the exception of [14]—a global spatially explicit product building on the methodology from [11]—works have primarily focused on geographic regions with high current multi-cropping extent, such as parts of China, India, and Indonesia. Regions such as the irrigated Western US, where the multi-cropping practice is less prevalent historically but has potential to increase under a changing climate, are largely ignored. Additionally, crop diversity in the irrigated Western US is very high (with hundreds of crops) compared to the handful of crop types typically referenced in existing studies. It is unclear if the methodology used in existing works will translate well into monitoring tools for areas of high crop diversity.

The typical methodologies used in quantifying cropping intensities are rule-based. They use a vegetation index (VI) time-series, apply threshold rules to identify peaks and troughs or starts and ends of the season, and count the number of crop cycles. Please note that by *time-series*, we refer to the sequence of VI over a given year. Given that these VI thresholds can vary by crop and region, some modifications to generalize the process are considered. This includes standardization practices that replace the direct use of a VI time-series with standardized values [11,14]. Additional enhancements to disregard spurious growth cycles are sometimes considered. For example, spectral indices indicative of bare soil such as the Land Surface Water Index (LSWI) are used to double-check the starts and ends of the season [10,12], especially when water-logged rice cropping systems are involved. A minimum crop-cycle-length constraint is also applied in some cases [11,14]. A key advantage of these methods is that a training dataset is not necessary.

While machine learning (ML) model applications—which require ground-truth datasets—are ubiquitous in applications like crop mapping, they are relatively rare and recent in crop intensity mapping [15,16,17,18,19,20]. However, these methods can be potentially helpful for learning important nuances in high-crop-diversity environments where vegetation thresholds and cycle lengths vary significantly by crop, soil characteristics, and farming practices (e.g., herbicide applications). Advantages of ML models are as follows: (i) they will be more accurate as the training set grows, (ii) they are more robust to rare or erroneous cases, and (iii) these methods learn mostly from frequent instances. The challenge in exploring ML methods is lack of ground-truth data (spatially explicit labeled cropping intensity) across a range of cropping systems for training the models. While all labeling exercises are time- and labor-extensive, some applications, such as the single-crop versus multi-crop distinction, are prohibitively so. For example, labeling images with irrigation canals or apples on a tree can be performed by researchers familiar with the topic by investigating the image once directly, with relatively minimal training. In contrast, labeling fields as single- or multi-cropped requires hiring enough personnel to visit fields multiple times within a season, which quickly becomes time- and cost-prohibitive.

**Figure 1:**
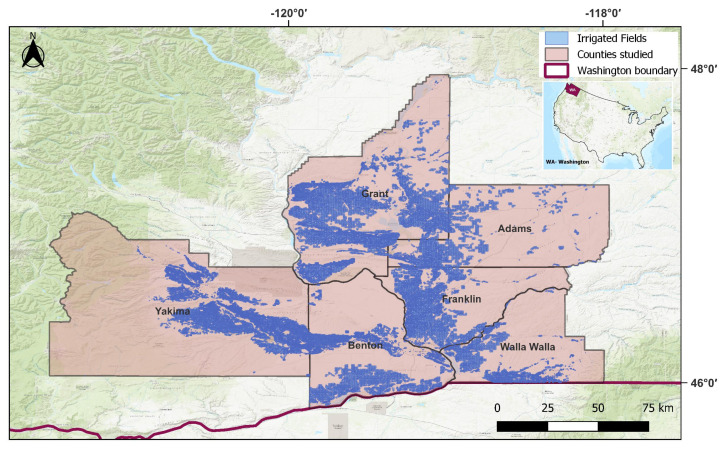
Case study region: eastern Washington State in the Pacific Northwest US. We focus on five counties with anecdotal evidence of double-cropping; Adams, Benton, Franklin, Grant, Walla Walla, and Yakima.

Our objective is to address the gap in field-scale single- and multi-cropped land-use data availability and monitoring capability in regions with high crop diversity. We address this by (a) labeling a ground-truth dataset in a relatively low-cost and scalable manner, (b) developing and evaluating ML classification methods for single- and multi-cropped fields that are crop-agnostic and at a field scale, and (c) comparing the performance of the ML models with the simpler rule-based method that is typically used. We consider a suite of ML models, but a novelty is the consideration of the time-series signature as an image and undertaking a deep learning image classification. Our hypothesis was that, given the fact that the shape of the time-series curve contains highly relevant information, image classification should work well. By *low cost*, we mean the relatively lower cost in terms of time and labor needs compared to drive-by/windshield surveys, which are prohibitively expensive for our use case and not scalable to large areas. Finally, we will apply our best-performing method to the irrigated agricultural extent of eastern Washington State in the Pacific Northwest US (Figure 1) as a case study to predict all fields as single- or double-cropped. Analysis of the predictions will help understand the capability of using this as a long-term monitoring tool for multi-cropping extent. In this regional context, multi-cropping is essentially double-cropping, and the rest of the paper uses this more specific terminology.

Our study area is located in the Upper Columbia River basin and the Yakima River basin of Washington State. This region is characterized by an arid climate with hot, dry summers and moderately cold, dry winters with significant spatial variability in climatic and topographic characteristics. The average annual precipitation ranges from 164 mm to 992 mm. The ground elevation ranges from 81 m to 3739 m. The region grows a wide variety of crops, most of which are high-value agricultural produce. This region is a good test-bed for several reasons. Even though double-cropping is practiced, single-cropping dominates, posing a challenge for identifying double-cropped fields. It is located in the northern latitudes, and as the climate warms and the growing season lengthens, conditions could become more conducive for double-cropping. Additionally, it relies on surface water for irrigation, and there are important field-scale water management challenges associated with increased double-cropping, necessitating spatially explicit information. This relates not just to the magnitude of additional irrigation water that might be needed but also to the timing. Water law in the states in the Western US generally specifies that water rights are associated with a specific *place and season of use*  regulatory burden on the water rights holder (farmer or irrigation district) and the managing agency (e.g., Washington State Department of Ecology) to modify the water rights specifications accordingly. This is a time- and cost-intensive process as changes need to be verified under the stipulations of the Western Water Law and Prior Appropriation Doctrine [21,22].

While recent cropping-intensity studies have leveraged data from the Sentinel mission—providing a 10 m spatial resolution with a 5-day revisit [23], we intentionally utilize data from the Landsat mission [24,25] at a 30 m spatial resolution and an 8-day (when two concurrent Landsat products are combined) revisit. This allows future leveraging of the Landsat time-series going back several decades—invaluable for understanding the trends, drivers, and impacts of adopting double-cropping. One might decide to use a harmonized Landsat and Sentinel product (or compare the performance of using only Landsat against such products) if focus is just on the current double-cropping extent.

For a low-cost labeling process that does not involve drive-by surveys multiple times within a season, we leverage satellite imagery and the knowledge of regional experts who are familiar with the cropping systems and fields. While this labeled dataset may not be as good as that from a drive-by survey, we expect that it will provide an adequate policy-relevant level of accuracy for identifying single- versus double-cropped extent. We develop and evaluate four ML models, and compare them with a traditional rule-based method that does not require ground-truthing to assess the importance of the labeling process. This is an important result for applying this method to other crop-diverse regions.

The double-cropping monitoring products that come out of this research should have broad global relevance, as cropping intensity is a global issue connected to food security and water-resource management and the interest in developing global-scale products [14]. It can inform many policy questions, for example, those related to meeting several of the UN 2030 Sustainable Development Goals [26], for which remote sensing applications are expected to be important [27]. Additionally, insights from this case study can be applied more broadly to the development of land-use datasets under the challenging contexts of prohibitively expensive costs in developing ground-truth datasets, which is a ubiquitous challenge across the globe.

## 2. Methods and Data

**Figure 2:**
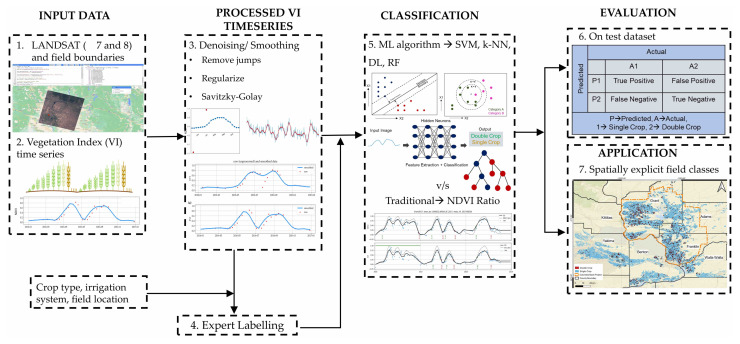
The overall workflow used for classifying the fields, from fetching the data to the final classification approaches, is provided here. First, we read satellite images on the Google Earth Engine (GEE), then compute vegetation indices, and then remove noise and smooth their time-series. These steps are followed by creation of ground-truth dataset, and finally, we train ML methods to classify them.

The overall workflow components described in this study are summarized in Figure 2. We first compute the field-scale smoothed NDVI time-series. These data, along with some additional information, are provided to the expert labeling process that creates the labeled ground-truth dataset; the information provided includes the field’s location on Google Maps so the experts have a sense of which field they are labeling, the date of data recording, the irrigation type, and the NDVI of the field in the year prior and after a given target year to provide a better picture of farming practices such as fall planting in the prior year or crop rotation practices. Classification models are then trained on the NDVI data and evaluated with the test set. Crop-specific summaries are also created for the test set. Finally, the models are applied over the full study area to obtain annual spatially explicit maps of double-cropping.

Our analysis is a field-level rather than pixel-level analysis because that is the decision scale and because pixel-level analysis has challenges related to spatial correlations and within-field differences in classes across pixels [28]. Therefore, this falls under the class of object-based analysis.

### 2.1. Input Datasets

The input data include a time-series of a commonly used VI—Normalized Difference Vegetation Index (NDVI) [29,30]—generated from multi-spectral observations at a 30 m spatial resolution from 2015 to 2018 (Table 1) from Landsat 7 and 8. We did not harmonize the Landsat 7 and 8 data. While [31] demonstrated that there is about a 5% bias between these datasets, we are interested in the overall shape of the curve, and the biases would get smoothed out with the data processing we describe below. With two concurrent missions running at a staggered temporal resolution of 16 days, we obtain a combined finer temporal resolution of ∼8 days—important for capturing the quick succession of harvest and planting cycles in double-cropped systems. A field-scale NDVI time-series is obtained by averaging values across all pixels contained within a field. More details of the time-series construction are provided in Section 2.3. We would like to note that we did explore the Enhanced Vegetation Index (EVI) that address saturation issues with NDVI but found no improved performance and do not report results with this index in this paper.

Given that our model development and application are intended for the field scale, we use the Washington Department of Agriculture’s (WSDA) Agricultural Landuse Geodatabase [32] to identify fields for our ground-truth data and broader mapping. This dataset was created via drive-by surveys by WSDA covering approximately a third of the state each year. That is, sections of the study area (Figure 1) were surveyed at least once over a three-year period. Each year’s dataset has the field boundary, crop type, irrigation system, and survey date. The crop type recorded is just the main crop that was observed at the time of the annual drive-by survey. That is, information on double-cropping is not present in this dataset, as that would require multiple drive-by passes of the same field each year.

To create a ground-truth set we only consider fields that were surveyed in the year under consideration so that the field boundaries and crop types are accurate (see Section 2.2 for the labeling process). We used data from the years 2015 to 2018, which resulted in 44,850 fields. Fields smaller than 10 acres were removed from the analysis for the following reasons: Our case study was conducted in parts of the US where field sizes tend to be larger in the absence of smallholder farms. However, smallholder farms are common in other parts of the world and in transferring the proposed methods to those regions the input data source might become critical. In these contexts, a 1-acre farm would have fewer than 5 Landsat pixels and field-scale vegetation indices averaged from a handful of pixels can have biases. Therefore, utilizing high-spatial-resolution satellite products—such as Sentinel-2 or commercial satellite imagery—to develop the VI time-series for the model will become important. However, this comes at the cost of not being able to develop cropping-intensity time-series that go back in time for decades like the Landsat-based products. Moreover, small fields can suffer from edge effects which result in a noisy VI signal. These smaller fields comprise less than 8% of the total area that we consider and are rarely double-cropped according to the experience of our experts. Therefore, their exclusion will have a minimal impact on our analysis. We also filtered out crop types with minimal irrigated acreage (less than 2874.57 acres—11.63 k$m^{2}$) in the study area. We selected 10% of the fields from each crop for labeling and added additional fields by crop as necessary to ensure that we have at least 50 fields per crop (if available) in the labeled ground-truth dataset. The fields were sampled from each crop individually with stratified sampling following best practices suggested by Stehman and Foody [28].

**Figure 3:**
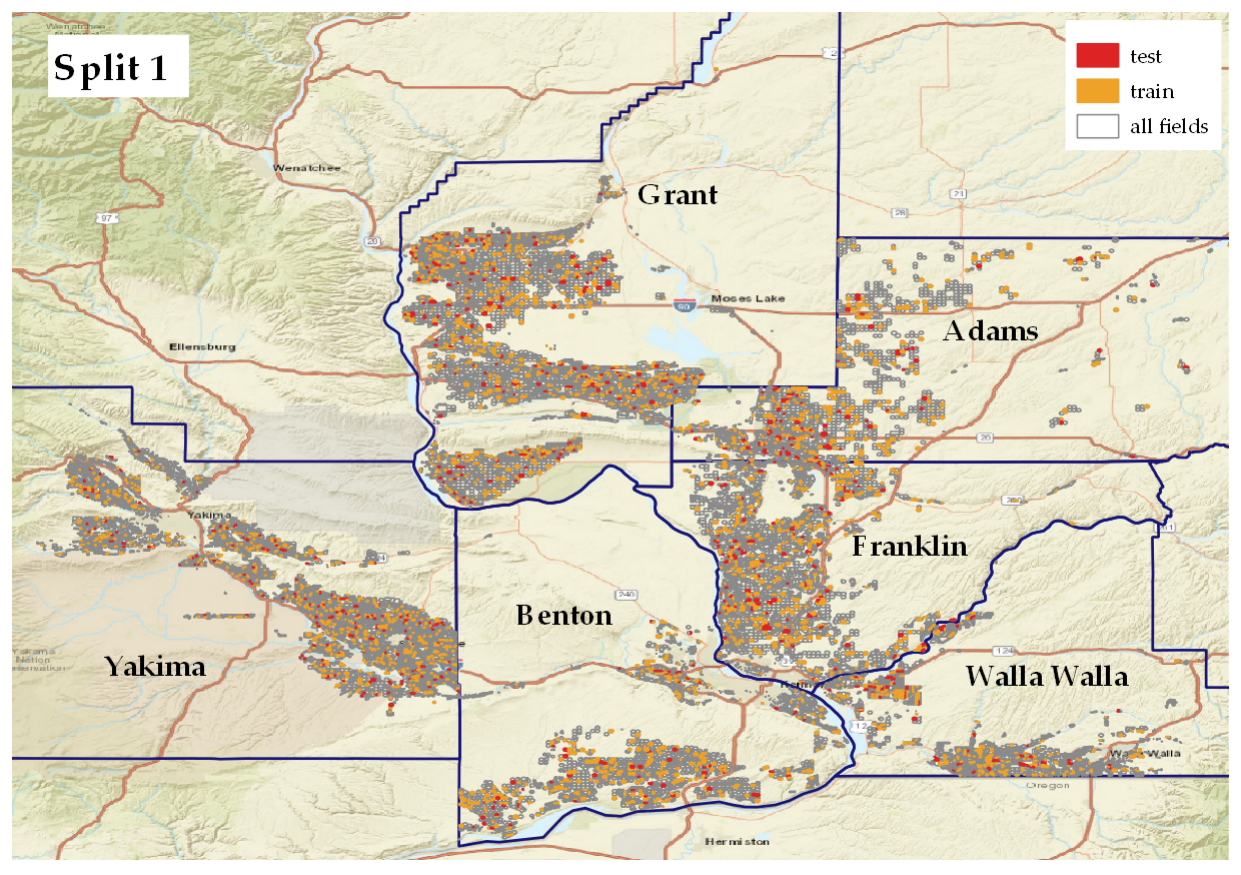
Training and test sets both cover the entire region of interest. They are geographically from the same distribution. Fields in the train set are colored in yellow, and test set is presented in red color. This figure shows one of the splits out of six. All splits are shown in Figure A2.

Figure 3 shows the distribution of the ground-truth set. The yellow dots are the fields used for training, and the red dots are the fields in the test set. As we can see, the fields are spread uniformly across the study region. For the final model application, we covered the entire irrigated extent in our counties of interest (Figure 1).

### 2.2. Expert Labeling Process

We collaborated with three individuals with extensive knowledge of cropping patterns in the region of study based on considerable time on the ground directly observing production. These individuals are professionals with university extension and governmental agricultural agencies (McGuire and Waters are county extension professionals from our region of interest, and Beale initiated and currently manages WSDA’s agricultural land-use mapping efforts. All experts have strong familiarity with fields, cropping practices, and growers in the region). Such experts are present in many agriculturally intensive production regions, which adds to the repeatability of our approach to other areas. Using multiple labelers aligns with the recommendation from [28] which states “protocols should be in place to monitor and evaluate the quality of the reference data and results. An example of quality assurance would be to evaluate consistency of reference class labels obtained by different interpreters” and is practiced by other researchers (e.g., [18]).

We had a three-step process for interacting with the expert panel. First was a meeting that allowed for a more open-ended discussion to identify crops and regions most associated with double-cropping, the timing of planting and harvesting of the first and second crops, and potential complications such as cover crops or hay crops that are harvested multiple times in the growing season. Such open-ended meetings are valuable for narrowing the scope of the analysis, although it is important to not allow existing perceptions that may be partially incorrect to influence the analysis. In our case, we took a nuanced position to cover-crops. While cover-crops are generally not considered a double-crop, since production is not harvested, we made an exception for the yellow-mustard cover-crop and categorized it as a double-crop because our interest is in the water footprint. Other cover-crops are planted very late in the season, requiring minimal or no irrigation. In contrast, yellow mustard is planted early enough to require significant irrigation. A research question focused on the total production of food and fiber, but not on resource use intensity, may result in a different categorization. It is also important to recognize that it may not be possible to differentiate a double-crop from a mustard cover-crop given the expected similarities in their time-series signatures of VIs.

To provide some additional context on the type of issues that may be discussed at this phase, the following are examples of topics raised: Certain crops (e.g., buckwheat, beans, peas, sweet corn, seed crops, sudangrass, triticale in some regions, and alfalfa) are more prevalent in double-cropping systems, and therefore, it would be good to know the crop type for the labeling process. Certain irrigation systems (e.g., flood) are not typically seen in double-cropped fields and the irrigation system would also be useful information for the labeling process. Additionally, double-cropping is prevalent in some areas and with some growers, and having a mapped location of the field will assist the expert in labeling by leveraging their local knowledge. In a double-cropping system, the second crop is typically planted by early August at the latest, and any indication of a second planting much after that is either a fall-planted crop for the following year or a cover-crop which should be considered a single-crop system for our purposes.

The second phase of the expert panel interaction was asynchronous labeling. For each field, we provided the VI time-series, the crop type, the irrigation system, and a Google Map link to the field’s location in a Google Form. The form had five choices for labels: “single-crop”, “double-crop”, “mustard crop”, “either double- or mustard crop”, and “unsure”. Before finalizing the ground-truth set to be sent to the expert panel, four other team members labeled easy-to-categorize crops—including crops that were clearly single-cropped from a clean VI time-series, as well as perennial tree crops and berries which we know cannot be double-cropped—and checked for agreement across team members. The remaining observations, which constitute the more difficult cases, were labeled by experts asynchronously with each field labeled by at least two experts.

We flagged the fields where the experts were not in agreement and resolved discrepancies during a final synchronous meeting. Some challenging discrepancies could not be resolved, and 15 fields were left as unsure and omitted from our dataset. The final labeled dataset consists of 3160 fields across four years (2015–2018) and is representative of 63 crops (Table 2) and five counties (Table 1). The labeling process was guided by more information than just the VI time-series, which is the only training input to the models.

### 2.3. Vegetation Index Time-Series

Crops have distinct spectral reflectance signatures related to their growth and phenological stages. In remote sensing applications, this is typically captured via VIs that capture the growth condition, phenology, and canopy cover of plants as the season progresses. While many VIs exist, a commonly used one in agricultural contexts is the NDVI [11,13,33], which is calculated from spectrometric reflectances in the red and near-infrared bands (Equation (1)). NDVI steadily increases after planting and decreases with senescence leading to harvest. We used the Google Earth Engine (GEE)—a geospatial processing platform that helps access the large suite of publicly available satellite imagery—to obtain field-scale NDVI time-series. We used Landsat Level 2, Collection 2, Tier 1 products (LE07/C02/T1_L2 [34] and LC08/C02/T1_L2 [35]) with atmospherically corrected surface reflectance bands.

(1) $$N\;D\;V\;I:=\frac{N\;I\;R-R}{N\;I\;R+R}$$

Satellite images are negatively affected by poor atmospheric conditions such as clouds or snow. These adverse effects typically lead to lower values of NDVI. As a part of data collection from GEE, we remove cloudy pixels and then compute the average field-scale NDVI using the remaining clean pixels. Taking the average across pixels is a standard and routine practice [36,37]. After fetching the data, we remove noise from the time-series to smooth the bumps and kinks that could throw off the detection of the crop growth cycle. There are numerous ways of smoothing, including weighted moving averages, Fourier and wavelet techniques, asymmetric Gaussian function fitting, and double logistic techniques. For a comparison of different smoothing methods, please see [38,39,40,41,42]. The performance of these methods depends on the data, the vegetation type, the data source, and the task at hand [39,40]. We use Savitzky-Golay (SG) [39,43], which is a commonly used polynomial regression fit based on a moving window. By varying the degree of polynomial and the size of window, one can adjust the level of smoothness.

Positive values of NDVI are indicative of greenness, and negative values represent the lack of vegetation where NDVI varies between −1 and 1.

The following steps are performed at the field scale for denoising:

1. **Correct big jumps.** The process of growing, and consequently, the temporal pattern of greenness cannot have abrupt increases within a short period of time. Therefore, if NDVI increases too quickly from time *t* to t+1, a correction is required. In such cases, we assume NDVI at time *t* is affected negatively and therefore we replace it via linear interpolation. In our study, we used a threshold of 0.018 as the maximum NDVI growth allowed per day; choosing this threshold makes sure that planting to peak canopy with high NDVI takes at least a month.
2. **Set negative NDVIs to zero.** Negative NDVI values are an indication of lack of vegetation. In our scenario, the magnitude of such NDVIs are irrelevant. The negative NDVIs with high magnitudes adversely affect the NDVI-ratio method for classification, and therefore, the negative values are set to zero. Assume there is only one erroneous NDVI value that is negative and large in magnitude, e.g., −0.9, while all other values are positive. Then, the NDVI-ratio method, which looks at normalized NDVI values, will have a large value in its denominator, and this single point affects the other points’ standardized values, which can throw off the NDVI-ratio method. Under this scenario, the NDVI-ratio at the time of trough will be pushed up, which leads to missing the harvest and re-planting in the middle of a growing year.
3. **Regularization.** In this step, we regularize the time-series so that the data points are equidistant. In every 10-day time period, we pick the maximum NDVI as representative of those 10 days. The maximum is chosen because NDVI is negatively affected by poor atmospheric conditions [44,45]. We utilize a 10-day regularization window as it is commonly used in the literature (e.g., [44,46,47]).
4. **Smooth the time-series.** As the last step, the SG filter is applied to the time-series to smooth them even further. SG filtering is a local method that fits the data with a polynomial using the least square method. The parameters used in this study are a polynomial degree of 3 and a moving window size of 7; i.e., a third-degree polynomial is fitted to seven data points.

### 2.4. Models

With the labeled data and VI time-series, we develop and evaluate five models to classify fields as single- or double-cropped. This includes the NDVI-ratio method used in prior related works [11,14] and four ML models: random forest (RF), support vector machine (SVM), k-nearest neighbors (kNN), and deep learning (DL).

**Train and test datasets.** A stratified splitting across crops (see Section 2.1) is applied to the ground-truth set six times to obtain six sets, each containing 80% training and 20% testing subsets. Within the 80% training data, a 5-fold cross-validation was used to train the ML models.

**NDVI-ratio method.** One widely used approach for detecting the start of the season (SOS) and end of season (EOS) is the so-called NDVI-ratio method of White et al. [48]. The NDVI-ratio is defined by

(2) $$N\;D\;V\;I_{ratio}(t):=\frac{N\;D\;V\;I_{t}-N\;D\;V\;I_{min}}{N\;D\;V\;I_{max}-N\;D\;V\;I_{min}},$$

where $N\;D\;V\;I_{t}$ is NDVI at a given time, $N\;D\;V\;I_{min}$ and $N\;D\;V\;I_{max}$ are the minimum and maximum of NDVI over a year, respectively. When this ratio crosses a given threshold, τ=0.5 [11,14], then there is an SOS or EOS event sometime close to the crossing. One pair of (SOS, EOS) event in a season is indicative of single-cropping, and two pairs are indicative of double-cropping. The following rules are applied under the NDVI-ratio method scenario:

1. If the range of NDVI during the months of May through October (inclusively) is less than or equal to 0.3, then this field is labeled as single-cropped. This step was motivated by the low and flat time-series of VIs exhibited by orchards during the visual inspection of figures.
2. Determine SOS and EOS by the NDVI-ratio method.
3. If an SOS is detected for which there is no EOS in a given year, we nullify the SOS. Such an event occurs for winter wheat for example. Similarly, if we detect one EOS over the year with no corresponding SOS, we drop the EOS and consider it as a single-cropping cycle. An example is winter wheat that is planted in the previous year.
4. A growing cycle cannot be less than 40 days.

**Machine learning models.** We build three statistical learning models—SVM, RF, and kNN—as well as a DL model for classification. Given that the kNN model computes the distance between two vectors (NDVI at multiple points in time), the vectors need to be comparable. Planting dates may vary by field, and therefore, the measurement for distance should take the time shift into account. This is accomplished by using dynamic time warping (DTW) as the distance measure for the kNN model. DTW was introduced by Itakura [49] in a speech recognition application and is now used in a variety of applications including agriculture [15,50]. It measures the distance between two time-series and takes into account the delay that may exist in one of the time-series; it warps time to match the two series so that the peaks and valleys are aligned.

For DL, we use the pre-trained VGG16 model provided by Keras and train only the last layer to avoid the overfitting problem. While the SVM, RF, and kNN models are provided with a vector of NDVI values as input, the DL model is provided with NDVI time-series images as inputs. This means that our approach to DL falls under the category of image classification. While time-series data are not usually analyzed in this manner [51], it allows for nuances in the shape of the time-series that other models may not capture, potentially resulting in better performance.

Given that there are fewer double-cropped than single-cropped fields, we oversampled the double-cropped instances (the minority class) to address the class imbalance in the dataset. Oversampling was performed by first identifying the number of majority class instances in the training set and then sampling (with replacement) from the minority class until its size reached a specified fraction of the majority class. The only tunable parameter in this process is the size ratio (size of minority class relative to the size of majority class). In our case, the optimal choice was 50%. Oversampling more than 50% led to lower performance. The problem, of course, is not the “imbalanced-ness”. The problem is that each class invades the space of the other class [52,53], and excessive oversampling contributed to lowering the performance in our case. Please note that there is no oversampling in the NDVI-ratio method, since there is no training involved and the rules in the NDVI-ratio method are pre-defined. These rules are applied to each individual field independent of other fields. In ML, however, all data points play a role in determining the shape of the classifier. Thus, oversampling is an attempt to tilt the weight in favor of the minority class.

The training process was optimized using 5-fold cross-validation. Details of search space (parameters to choose from) are in Appendix A.1. The models were implemented using the Python (*v3.9.16*) scikit-learn package (*v1.2.1*) for RF, SVM, and kNN. For the DTW metric used in kNN, dtaidistance *v2.3.9* was used. For DL, we used TensorFlow (*v2.9.1*) and Keras (*v2.9.0*) (data processing and visualization were performed using multiple Python packages. Scripts are accessible at https://github.com/HNoorazar/NASA_WWAO_DoubleCropping/tree/main).

**Accuracy assessment.** There are three accuracy metrics used and presented in this study. Overall accuracy (OA) is used during training and for optimizing the parameters and hyperparameters. For the testing phase, we report count-based overall, user’s, and producer’s accuracies (UA, PA), as well as their standard errors (SEs) following the methods described in [54], which accounts for the sampling strategy (e.g., stratifications).

Unlike OA, UA and PA provide information on errors associated with each specific class. The PA helps identify consistent under-representation of a class on a map while the UA indicates if a class is consistently mislabeled as another class. The PA (for the double-cropped class) refers to the fraction of true double-cropped fields that were predicted as double-cropped, and the UA refers to the fraction of predicted double-cropped fields that were actually double-cropped.

Given that we are interested in double-cropped areas, we also report the proportional area-based confusion matrix; however, we are unable to report SEs given that there is no available methodology to address this for object-based classification [28,55].

## 3. Results

In this section, we compare the performance of the ML models against each other and the NDVI-ratio method. We present crop-specific and regional summaries on the test dataset and apply the DL model to all the fields in the study region for specific years (Table 1) reflective of the “current” double-cropping extent.

### 3.1. Accuracy Statistics

In this section, we present the results of classifying the test sets with the trained models. The four ML models outperform the NDVI-ratio method with overall accuracies ranging from 95% to 99% as opposed to 89–91%(Table 3) across the six test sets. Individual model performance is provided in Table A2 in Appendix A.2. Additionally, the UA of the NDVI-ratio method is poor (46–54%), with significant over-prediction of single-crops as double-crops.

The DL model has the best overall performance, and the DL and SVM models have the best performance in identifying double-cropped fields (Table 3). The RF model has a relatively higher UA than PA unlike other models. The implication is that the RF model is less likely to mislabel single-crops as double-crops, while the DL and SVM models are less likely to mislabel double-crops as single-crops.

The standard errors of OA are between 0.1 and 0.8% and are fairly consistent across all models (Table 4). For the double-cropped fields, the range for standard errors of PA and UA are 0 to 5.4% and 1.2% to 6.4%, respectively, across all models. The DL model has a narrow range of standard error (0 to 2.5%) for PA (for double-cropped fields).

**Figure 4:**
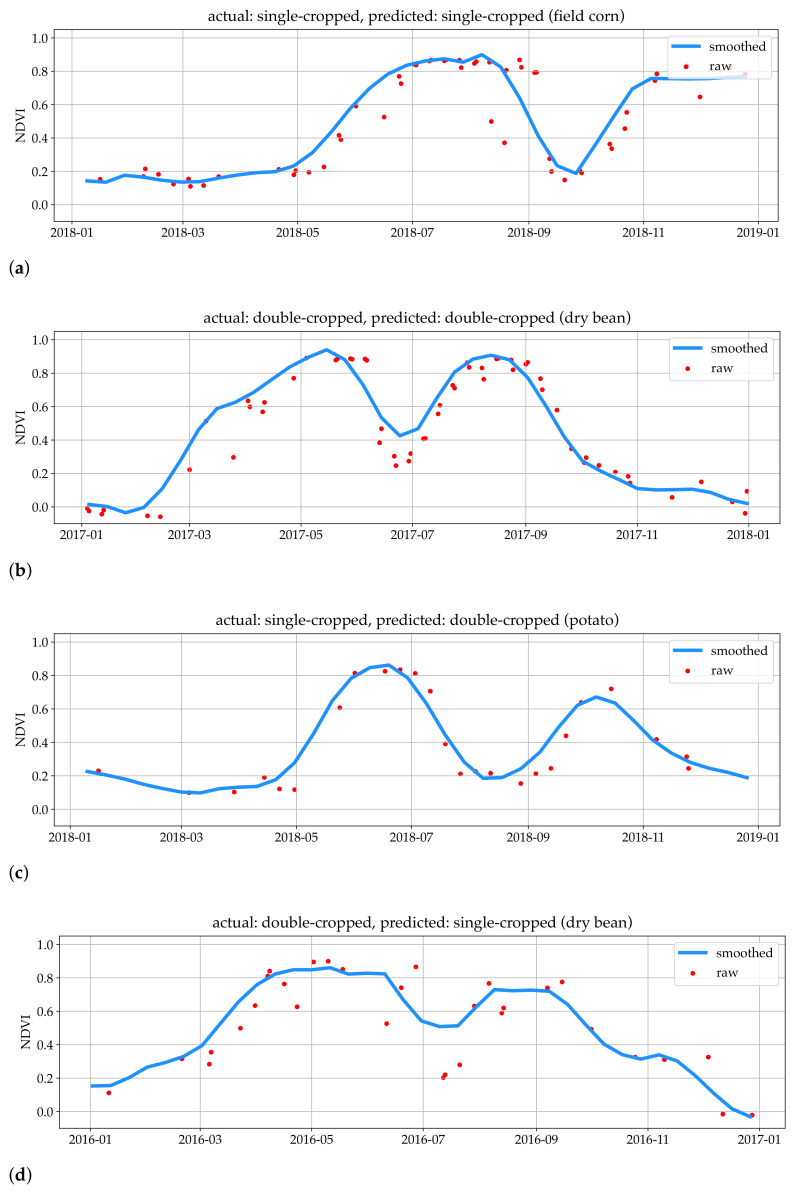
Examples of raw and smoothed time-series from each group in the confusion matrix for the DL column of Table 3. (**a**) shows a single cropped field. There is a late increase in NDVI which is a cover-crop or a winter-crop and thus this field does not count as a double-cropped field. The algorithm and the ground-truth label agree; (**b**) shows two clear maximums indicating a double-cropped event where the algorithm and the ground-truth labels are in agreement; (**c**) is an example of false double-crop detection. The second bump is associated with a cover crop; the increase and decrease in NDVI time-series are too late to have a second crop planted and harvested; (**d**) shows a double-cropped field that is mislabeled by the algorithm due to over-smoothing.

Figure 4 shows the NDVI time-series for four fields labeled by the DL model. Two fields are labeled correctly (Figure 4a,b) and two are labeled incorrectly (Figure 4c,d). For the field shown in Figure 4c, the model has missed the fact that the second crop’s cycle is a bit too late in the season (starting in September). In Figure 4d, where the model incorrectly labeled a double-cropped field as a single-crop, the noise in the raw data (red dots) seems to have resulted in over-smoothing (blue line), misleading the model.

While the above statistics are field-count-based, the confusion matrices for the proportional areas of single- and double-cropped extent are provided in Table A1 in Appendix A.2. The proportion area predicted as double-cropped by the DL models ranged from 11% to 14%, while the ground-truth area proportion ranged from 13% to 17% across the test sets. In our case, the OA from the area-based confusion matrix is similar to that from the count-based confusion matrix, providing confidence that the method can be used to estimate areas under single- and double-cropping. If these accuracies are very different, that can be indicative of errors being different for small versus large fields and point to the need to exercise caution in interpreting mapped area estimates. Note that these statistics must be interpreted with caution as our study is object-based (see the accuracy assessment in Section 2.4).

### 3.2. Fraction of Double-Cropped Acres by Crop

**Figure 5:**
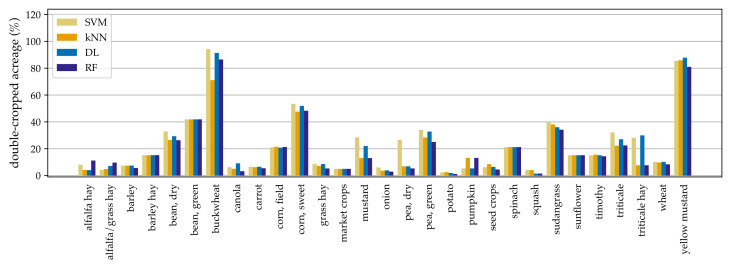
Fraction (in %) of each crop’s acreage that is classified as a double-cropped. This is based on the test dataset. Perennial crops such as apples, which cannot be double-cropped, would have bar heights very close to zero are not displayed in this figure for visual clarity.

In our initial meetings with the experts, the following were identified as common double-cropped crops: buckwheat, beans, peas, sweet corn, seed crops, sudangrass, triticale (in some regions), and alfalfa. Based on our crop-wise classification statistics on the test dataset (Figure 5), the crops with relatively higher fractions match this list, except for alfalfa. Alfalfa is a perennial crop with a 3- to 4-year cycle, where double-cropping is a possibility only in the final year of the cycle. Given this, our labeling in the training set skewed toward classifying it as a single-crop, and we may be underestimating the double-cropped acres for this crop.

The high percentages of buckwheat and yellow mustard are in agreement with the local knowledge that buckwheat is almost always double-cropped and that yellow mustard is primarily a cover crop following a potato crop. This is in contrast with the mustard crop type that is grown as a seed crop with a low double-cropped fraction. Moreover, perennial tree fruits and berries, such as apples and blueberries, cannot be double-cropped, and the model performs well in capturing them as single-crops (only 2 out of 8429 fields were incorrectly labeled). While all models are generally in agreement across crops, the differences across models are larger for some crops (e.g., triticale hay, mustard, and buckwheat).

### 3.3. Regional Summary and Spatial Distribution of Double-Cropped Fields

Over the six counties analyzed, around 10% of the total irrigated extent was double-cropped based on the best DL model. While we do not have the ground-truth data to compare with this overall number, it is in qualitative agreement with anecdotal expectations and local experts’ knowledge that the region has some but not a lot of double-cropped extent.

**Figure 6:**
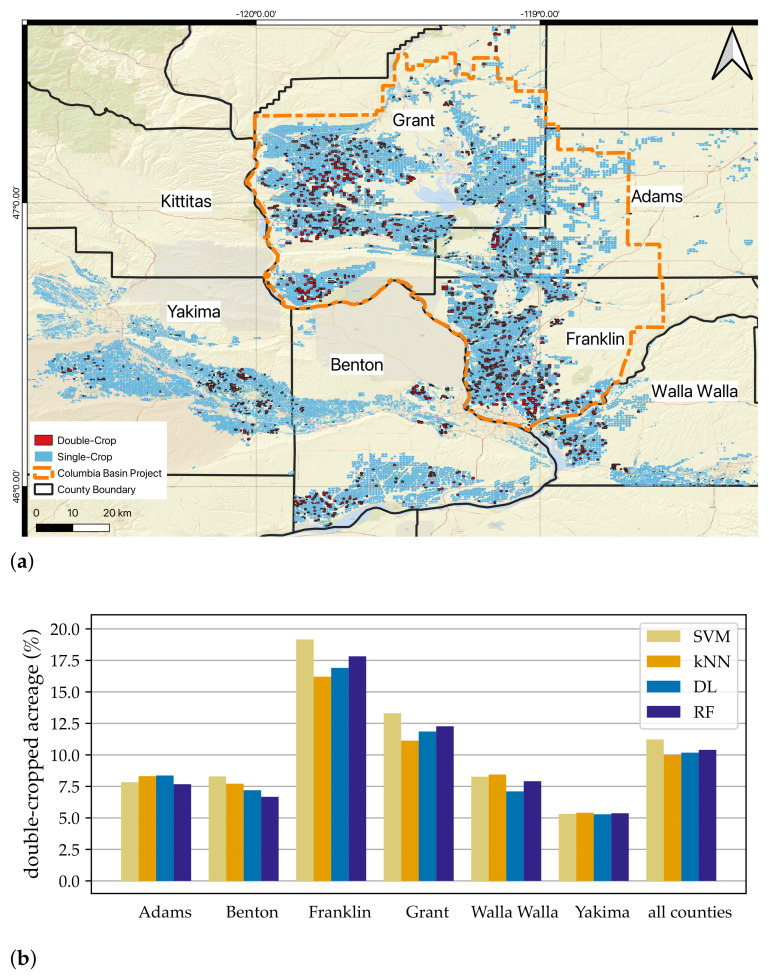
(**a**) The spatial distribution of classes of fields is presented in this figure alongside (**b**) the double-cropped area as percentages of total irrigated extent in each of the counties. The predictions of ML algorithms are in agreement with experts’ expectations; majority of double-cropped fields are in Franklin and Grant counties and Yakima has the lowest rate.

The majority of double-cropped fields are in the United States Bureau of Reclamation’s Columbia Basin Irrigation Project area, which covers parts of Franklin, Adams, and Grant counties. The lowest rate of adoption is in Yakima county (Figure 6a,b). This is also in agreement with the experts’ expectations and our knowledge of water rights security in the region [56,57]. Water use associated with growing two crops requires secure senior water rights, and the Columbia Basin Irrigation Project region has the water rights that can facilitate this. In contrast, a region like Yakima county has a large fraction of junior water rights holders for whom water rights restrictions may constrain the ability to adopt double-cropping.

## 4. Discussion

Two metrics indicate that our approach to identifying double- versus single-cropped fields works well and could be extended to other regions with crop diversity. The first is the high OA and PA of our DL model on the test sets. The second is the qualitative evidence that we are capturing the overall double-cropped crop types and spatial extent in line with experts’ expectations. Additionally, the DL model has fewer instances of mislabeling double-cropped as single-cropped and therefore higher PA and lower standard errors for the double-cropped class. For some applications, PA might be more important. For example, a policy maker interested in capturing the double-cropping extent for estimating the related water footprint will likely prefer a higher PA.

While the adjusted NDVI-ratio method (with augmented auxiliary algorithms) performed well in identifying double- and triple-cropped fields in another work [11], its performance was poor in our case with very low user’s accuracy (46-59%) and over-prediction of the double-cropped class. Note that this is not a direct comparison because (a) the geographic area is different, (b) Liu et al. [11] is a pixel-level analysis, while ours is object-based at a field scale, (c) we exclude cover-crops and focus on harvested crops, which is consistent with the definition of multi-cropping from a food security perspective, and the NDVI-ratio methods are unlikely to be able to make this distinction unless a season definition is included, and (d) our methods have a training process that allows the model to learn more nuances related to the shapes of the VI time-series compared to a simpler rule-based method. Therefore, while the difference in performance cannot be solely attributed to the diversity of crops in our region, it likely contributed to it. Each crop has different canopy cover and VI characteristics, likely rendering one threshold value to be inadequate in capturing this diversity.

A more direct comparison is possible with a recent global spatially explicit cropping-intensity product at a 30 m resolution [14]. We overlaid the global product with our study region and identified the overlapping area for comparison. Given significant missing data in the global product in one of our counties of interest (Yakima county), we excluded that county from the comparison. We aggregated the pixel-level cropping intensity from [14] to our field level to obtain the dominant intensity class. The resulting multi-cropped extent (primarily double-cropped) was ≈1015.76k$m^{2}$ compared to our best DL model’s estimate of ≈437.06k$m^{2}$. Therefore, while the global product from [14] is expected to be a conservative estimate in general, we find that it may be significantly overestimating the cropping intensity in our irrigated study area and perhaps across all of Western US irrigated extent. We hypothesize that this could be due to several reasons. First, the methodology of [14] is based on an NDVI-ratio process which, as we demonstrated, does not work well and overestimates double-cropping in our highly diverse cropping environment. Additionally, our comparison highlighted that the product in [14] seems to have erroneously classified a significant acreage of perennial crops (e.g., tree fruit, berries, alfalfa) as double-cropped instead of single-cropped. This highlights that there is an opportunity to integrate ML methods that can be trained to capture additional nuance and regional insights into the global products to enhance their utility, especially in regions such as ours that are not well covered and tested in the cropping-intensity mapping literature.

We acknowledge that operationalizing this as a global product is more complex than rule-based methods, whose simplicity is a strength for broad global-scale applications. But the benefits of trained models can be substantial in complex contexts. Operationalizing an ML-based global product would need (a) further exploration of ML approaches in crop intensity mapping, (b) a concerted effort to curate a global-scale public training dataset that is accessible to researchers across the world including metadata about regional cropping systems and crop cycles which would be relevant, and (c) significant coordination to bring in local expertise for labeling. This is not an easy task and as regional datasets that are more reliable start to develop perhaps where available and can be integrated into global products that are based on more scalable rule-based methods.

The expert labeling process we describe here is much less burdensome than extensive drive-by surveys for land-use labeling. The challenge is in identifying the right set of local experts for the application under consideration. At least in the agricultural context of the US, the Land Grant University County Extension system has an established network of local experts that are familiar with the area, the fields, the growers, and their practices and are an excellent source of knowledge that should be leveraged more in developing labeled datasets that can aid remote sensing applications. While the precision of a drive-by survey may not be achievable, useful information for informing policy can be developed as demonstrated in this work.

One aspect we have not thoroughly evaluated in the temporal scalability of results. Typically, this would be evaluated as a *leave-one-year-out* experiment. However, in our case, the dataset spans different regions in different years, making it difficult to isolate temporal scalability from spatial variability. Additionally, the relatively limited size of our ground-truth dataset poses further constraints. That said, we do not anticipate major issues with generalizing the model across years, unless there are extreme climatic anomalies—such as unusually warm or cold years—that significantly alter crop phenology. Such shifts could affect key assumptions in our ground-truth labeling, particularly regarding planting dates that distinguish double-cropping from cover-cropping. Additionally, in scenarios involving long-term climate trends, non-stationarity may gradually alter phenology. However, such shifts typically occur over several decades and generally result in week-scale changes which do not impact our assumptions as much. Given this, we believe the model should remain applicable across the past few decades and into the near future, but the temporal scalability issues should be borne in mind for applications.

This method has a number of potential extensions and applications. The first is extending the geographic scope of this work. While the model we have developed is publicly shared and can be directly applied, some additional labeling and retraining would likely be necessary, especially for regions where the cropping intensity can be larger than two crops in a year. Even in this scenario, our DL model can be used for transfer-learning approaches, which can shorten the training time. The models can be continually retrained and refined as new labeled data are created. While our ground-truth data collection and model development efforts focused on irrigated areas, the method is generic and should work for dryland regions as well, though this will need to be tested. Additionally, while our model development and application were at a field scale, which is the scale of decisions, the method can be directly applied at a pixel scale as well in areas where field boundary data are not easily available.

The second is to apply the proposed method and develop a historical time-series of double-cropped extent. This would allow a trend analysis that could be used to identify drivers of increased or decreased double-cropping including changing climatic patterns and agricultural commodity market conditions [58]. Further analysis would be needed to isolate the climatic drivers of trends, but the methods and dataset developed here are an important first step in generating data that will facilitate these analyses. Climatic relationships, once established, can be extrapolated in a climate-change context to quantify the potential for increased double-cropping in the future. When coupled with a cross-sectional analysis of historical double-cropping trends across diverse regions, climatic thresholds at which double-cropping becomes infeasible (e.g., because the summers are too hot to double-crop) can also be identified. This will provide a realistic upper bound of the potential for increased double-cropping.

Finally, from a water footprint perspective, this spatially explicit dataset can be a valuable input to crop models (e.g., VIC-CropSyst [59]) to quantify irrigation demands under current and future conditions of double-cropping adoption. Existing studies on climate-change impacts on irrigation demands do not account for double-cropping, and this addition will address an important missing component in the climate-change-impacts literature. This can inform the planning efforts of various stakeholders, including irrigation districts planning for infrastructure management based on season length, water rights management agencies planning for change requests, and water management agencies planning to minimize scarcity issues or adjust reservoir operations.

There are several other policy questions and trade-offs that can be informed by utilizing such spatially explicit datasets (along with other data and models). For example, *how much of the growing global food demand can multi-cropping meet? Are the environmental outcomes of increased food production though intensification on existing land better or worse than outcomes from expanding agricultural land? What are the impacts of fertilizer inputs on the land or carbon sequestration potential?* As stewards of our natural resources, it is important for us to address the broad range of trade-offs that accompany any land-use change trends and equip ourselves with datasets and monitoring platforms that help address them.

## 5. Conclusions

The rule-based methods that are widely used to map cropping intensity did not work for our high-crop-diversity context. In contrast, ML models, especially the DL model, were able to learn crop-specific nuances and achieved overall accuracies of 96% to 99%, as well as user’s and producer’s accuracies of 74 – 92% and 83 – 93%, respectively, for the double-cropped class, while also identifying double-cropping in the right crop types and locations based on expert knowledge. Given that these models require labeled ground-truth data, low-cost labeling efforts that leverage satellite imagery and experts’ knowledge will be key to expanding the availability of such land-use datasets at global scales.

Given that these labeling efforts are likely to be local/regional endeavors, a centralized mechanism to publicly share these labeled datasets will allow the research community to make continued advancements in modeling and monitoring the environment via remote sensing approaches. The availability of these datasets and monitoring platforms is critical for answering key policy-relevant questions related to food security and natural resource sustainability.

## Figures and Tables

**Table 1:** Field boundaries filtered for the different counties based on the surveyed date.

**county**	Adams	Benton	Franklin	Grant	Walla Walla	Yakima
**survey year**	2016	2016	2018	2017	2015	2018

**Table 2:** Crop varieties in the ground-truth set used for training the models. The pair of numbers for each crop indicates the number of fields and the total area in acres, respectively.

alfalfa hay (43–2564)	fescue seed (9–533)	poplar (44–2445)
alfalfa seed (23–1274)	grape, juice (71–1926)	potato (185–14,989)
apple (543–13,370)	grape, wine (128–3603)	pumpkin (9–272)
apricot (16–326)	grass hay (46–1492)	ryegrass seed (8–513)
asparagus (23–1150)	grass seed (34–2039)	sod farm (15–796)
barley (11–575)	hops (120–3040)	squash (9–450)
barley hay (12–636)	market crops (10–181)	sudangrass (37–1704)
bean, dry (71–3482)	medicinal herb (8–225)	sugar beet (10–748)
bean, green (44–1989)	mint (34–1485)	sugar beet seed (17–1430)
blueberry (24–1004)	mustard (2–138)	sunflower (7–387)
bluegrass seed (42–3958)	nectarine/peach (18–467)	sunflower seed (29–1185)
buckwheat (41–2695)	oat hay (5–104)	timothy (70–4848)
canola (27–2568)	onion (44–3108)	triticale (18–862)
carrot (45–2687)	onion seed (8–333)	triticale hay (17–538)
carrot seed (17–644)	orchard, unknown (9–287)	watermelon (10–462)
cherry (106–2350)	pasture (141–4403)	wheat (207–14,151)
corn seed (16–516)	pea seed (1–15)	wheat fallow (42–3239)
corn, field (316–16,558)	pea, dry (26–1180)	wildlife feed (27–1065)
corn, sweet (61–4975)	pea, green (40–3248)	yellow mustard (20–1622)
fallow (31–692)	pear (31–594)	
fallow, idle (22–528)	pepper (4–297)	
fallow, tilled (21–837)	plum (4–64)	

**Table 3:** Classification from the test sets. The ranges correspond to values from the six test sets. User’s and producer’s accuracies (UA and PA) reported in this table are for the double-cropped class. Table A2 in Appendix A.2 shows the results separated by each test set.

GT	pred.	SVM	DL	kNN	RF	NDVI-R
**1**	**1**	556–563	555–568	554–562	562–571	517–536
**2**	**2**	46–55	49–55	43–48	41–44	43–48
**1**	**2**	10–17	5–18	11–19	2–11	37–56
**2**	**1**	4–13	4–10	11–16	15–18	11–16
**# err**		15–30	9–25	25–33	20–29	52–67
**OA**		95–98%	96–99%	95–96%	95–97%	89–93%
**UA**		73–84%	74–92%	70–80%	79–95%	46–59%
**PA**		78–92%	83–93%	73–81%	69–75%	72–83%

**Table 4:** Range of accuracy metrics for ML models across the six splits of ground truth: overall accuracy (OA), user’s accuracy (UA), producer’s accuracy (PA), and standard errors (SEs).

Parameter	SVM	DL	kNN	RF
**OA**	95–98%	96–99%	95–96%	95–97%
**OA SE**	0.2–0.8%	0.1–0.5%	0.2–0.6%	0.2–0.7%
**UA double**	73–84%	74–92%	70–80%	79–95%
**UA double SE**	2.1–5.5%	1.4–5.2%	3.2–5.7%	2–6.4%
**PA double**	78–92%	83–93%	73–81%	70–75%
**PA double SE**	1.6–5.3%	1.2–2.5%	2.2–3.2%	2.4–5.4%

**Figure A1:**
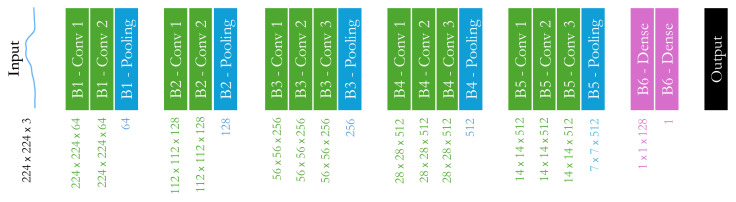
VGG16 is the deep learning structure used in this manuscript. By setting include_top=False, we remove Block 6 with three dense layers at the end and replace and train only two layers instead. The first five blocks are pre-trained and provided by the Keras library of Python.

**Figure A2:**
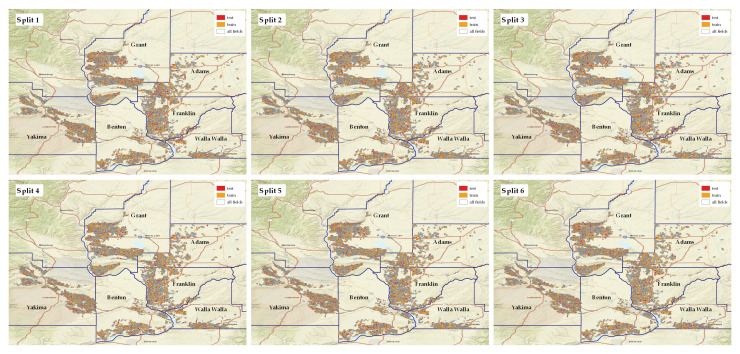
All of the six splits of ground-truth set into training and test sets.

## Data Availability

The datasets generated during and/or analyzed during the current study are available from the corresponding author on reasonable request.

## Abbreviations

    The following abbreviations are used in this manuscript:

DL	Deep Learning
DTW	Dynamic Time Warping
EOS	End of Season
GEE	Google Earth Engine
GT	Ground Truth
kNN	k-Nearest Neighbors
ML	Machine Learning
NDVI	Normalized Difference Vegetation Index
NDVI-R	NDVI-ratio
OA	Overall Accuracy
PA	Producer’s Accuracy
RF	Random Forest
SE	Standard Error
SG	Savitzky-Golay
SOS	Start of Season
SVM	Support Vector Machine
UA	User’s Accuracy

## Appendix A

### Appendix A.1. Details on ML Methods

The cost function that is minimized in the ML models is overall accuracy. The parameters tried for each specific model to obtain the best model are listed below.

DL Since our ground-truth set is small and the shapes of the time-series are not complicated, we used a pre-trained network (transfer learning) from the keras package. Our choice of architecture is VGG16, whose slightly modified structure is shown in Figure A1. The modification is in the last fully connected layers, where we have two dense layers as opposed to the original three. This architecture consists of 13 convolutional layers, five max-pooling layers, and two dense layers at the end; (Block 1) the first two convolutional layers consist of 64 filters each, with kernels of size three-by-three with the same padding applied in these layers. The size of filters and the paddings are identical in all of the convolutional layers. These two layers are followed by a max-pool layer, where the filter is of size two-by-two and the stride size is also two. The max-pool layers throughout the network are identical in their sizes and strides. (Block 2) There are two convolutional layers with 128 filters, followed by another max-pool layer identical to the one in Block 1. (Block 3) Three convolutional layers, each of which consists of 256 filters, followed by a max-pool layer. (Block 4) Three convolutional layers, each of which consists of 512 filters, followed by another max-pool layer. (Block 5) Once again, Block 4 is repeated, followed by Block 6, which consists of two densely connected layers.
  Block 6 is where VGG16 is adjusted in this manuscript; we have only two dense layers as opposed to the original three. The first five blocks are pre-trained and are provided by Keras. The activation function for all layers is ReLU except for the last layer, which is a softmax layer with sigmoid activation, since we have a binary classification problem at hand.

**kNN** The parameters used for optimizing kNN are the number of neighbors and the weight parameters;
  n_neighbors∈ {2, 4, 6, 7, 8, 9, 10, 11, 12, 13, 14, 15, 16, 17, 18, 19, 20},
  weights∈{uniform, distance}

RF For the random forest, the parameters we searched over are
  criterion ∈{gini,entropy}
  max_depth∈ {2, 4, 6, 8, 9, 10, 11, 12, 13, 14, 15, 16, 18, 20 }
  min_samples_split∈ {2, 3, 4, 5 }
  max_features∈ {sqrt, log2, None},
  class_weight∈ {balanced, balanced_subsample, None}
  ccp_alpha∈ {0, 1, 2, 3},
  max_samples∈ {None, 1, 2, 3, 4, 5}
SVM As part of the SVM technique, we applied a grid search over the following set of parameters:
  Regularization parameter c ∈ {5, 10, 13, 14, 15, 16, 17, 20, 40, 80},
  The kernel functions tried are {linear, polynomial (polynomial of degree 3), rbf (rbf is radial basis function), sigmoid},
  class_weight∈ {balanced, uniform}.

### Appendix A.2. All Splits of Ground-Truth Set

We divided the ground-truth set into 80-20% splits for training and testing for a total of six different times. This helped us to check if the test set in the main body of the manuscript was accidentally an easy set to classify, or perhaps the training set was rich enough to capture all the different patterns.

We report the different split results below. We emphasize that the results below are the output of applying the trained model to the test sets. In other words, the test set is not used in the training process, and thus, a good result is not an indication of overfitting. These tables together show the variation that could occur due to changes in the training set.

Table A1 presents the area-based confusion matrix. We, however, refrain from presenting the accuracies based on the area since “in terms of the proportion of the area of the map that is incorrectly classified, an error for a large polygon has obviously more impact than an error for a smaller one” [60].

**Table A1:** Confusion matrices based on field-area ratios for all 6 different splits of the ground-truth dataset.

GT	pred.	SVM	DL	kNN	RF
**Train-Test Split 1**					
1	1	0.85	0.86	0.85	0.86
2	2	0.12	0.09	0.1	0.12
1	2	0.02	0.02	0.02	0.01
2	1	0.01	0.03	0.03	0.01
**Train-Test Split 2**					
1	1	0.82	0.83	0.82	0.82
2	2	0.13	0.1	0.11	0.14
1	2	0.02	0.02	0.02	0.03
2	1	0.02	0.05	0.04	0.01
**Train-Test Split 3**					
1	1	0.8	0.82	0.8	0.79
2	2	0.12	0.12	0.12	0.15
1	2	0.04	0.02	0.03	0.05
2	1	0.04	0.05	0.04	0.01
**Train-Test Split 4**					
1	1	0.82	0.83	0.82	0.82
2	2	0.11	0.1	0.1	0.13
1	2	0.03	0.02	0.03	0.03
2	1	0.04	0.05	0.05	0.02
**Train-Test Split 5**					
1	1	0.83	0.85	0.82	0.83
2	2	0.13	0.1	0.11	0.13
1	2	0.03	0.01	0.03	0.03
2	1	0.02	0.04	0.04	0.01
**Train-Test Split 6**					
1	1	0.82	0.83	0.82	0.82
2	2	0.11	0.11	0.11	0.12
1	2	0.03	0.02	0.03	0.03
2	1	0.05	0.04	0.04	0.04

**Table A2:** Classification results from the test sets. User and producer accuracies reported in this table are for the double-cropped class. These statistics are count-based.

GT	pred.	SVM	DL	kNN	RF	NDVI-R
**Train-Test Split 1**						
**1**	**1**	562	568	559	565	540
**2**	**2**	55	55	48	44	49
**1**	**2**	11	5	14	8	33
**2**	**1**	4	4	11	15	10
**#err**		15	9	25	23	43
**OA**		0.98	0.99	0.96	0.96	0.93
**UA**		0.84	0.92	0.77	0.85	0.59
**PA**		0.92	0.93	0.81	0.71	0.83
**Train-Test Split 2**						
**1**	**1**	563	561	562	566	536
**2**	**2**	51	54	45	41	44
**1**	**2**	10	12	11	7	37
**2**	**1**	8	5	14	18	15
**#err**		18	17	25	25	52
**OA**		0.97	0.97	0.96	0.96	0.91
**UA**		0.84	0.82	0.80	0.85	0.54
**PA**		0.86	0.92	0.76	0.69	0.74
**Train-Test Split 3**						
**1**	**1**	556	555	554	564	517
**2**	**2**	46	54	45	42	48
**1**	**2**	17	18	19	9	56
**2**	**1**	13	5	14	17	11
**#err**		30	23	33	26	67
**OA**		0.95	0.96	0.95	0.96	0.89
**UA**		0.73	0.75	0.70	0.82	0.46
**PA**		0.78	0.92	0.76	0.71	0.81
**Train-Test Split 4**						
**1**	**1**	558	555	557	562	533
**2**	**2**	48	52	43	41	45
**1**	**2**	15	18	16	11	40
**2**	**1**	11	7	16	18	14
**#err**		26	25	32	29	54
**OA**		0.96	0.96	0.95	0.95	0.91
**UA**		0.76	0.74	0.73	0.79	0.52
**PA**		0.81	0.88	0.73	0.70	0.76
**Train-Test Split 5**						
**1**	**1**	561	557	554	571	523
**2**	**2**	54	54	45	41	43
**1**	**2**	12	16	19	2	50
**2**	**1**	5	5	14	18	16
**#err**		17	21	33	20	66
**OA**		0.97	0.97	0.95	0.97	0.89
**UA**		0.82	0.77	0.70	0.95	0.46
**PA**		0.92	0.92	0.76	0.70	0.72
**Train-Test Split 6**						
**1**	**1**	558	561	559	565	528
**2**	**2**	46	49	48	44	45
**1**	**2**	15	12	14	8	45
**2**	**1**	13	10	11	15	14
**#err**		28	22	25	23	59
**OA**		0.96	0.97	0.96	0.96	0.9
**UA**		0.75	0.80	0.77	0.85	0.5
**PA**		0.78	0.83	0.81	0.75	0.76
